# Current possibilities and future opportunities provided by three-dimensional lung ECM-derived hydrogels

**DOI:** 10.3389/fphar.2023.1154193

**Published:** 2023-03-09

**Authors:** Mehmet Nizamoglu, Janette K. Burgess

**Affiliations:** ^1^ University of Groningen, University Medical Center Groningen, Department of Pathology and Medical Biology, Groningen, Netherlands; ^2^ University of Groningen, University Medical Center Groningen, Groningen Research Institute for Asthma and COPD (GRIAC), Groningen, Netherlands; ^3^ University of Groningen, University Medical Center Groningen, W.J. Kolff Institute for Biomedical Engineering and Materials Science-FB41, Groningen, Netherlands

**Keywords:** extracellular matrix, fibrosis, *in vitro* models, collagen, chronic lung disease, biomechanics

## Abstract

Disruption of the complex interplay between cells and extracellular matrix (ECM), the scaffold that provides support, biochemical and biomechanical cues, is emerging as a key element underlying lung diseases. We readily acknowledge that the lung is a flexible, relatively soft tissue that is three dimensional (3D) in structure, hence a need exists to develop *in vitro* model systems that reflect these properties. Lung ECM-derived hydrogels have recently emerged as a model system that mimics native lung physiology; they contain most of the plethora of biochemical components in native lung, as well as reflecting the biomechanics of native tissue. Research investigating the contribution of cell:matrix interactions to acute and chronic lung diseases has begun adopting these models but has yet to harness their full potential. This perspective article provides insight about the latest advances in the development, modification, characterization and utilization of lung ECM-derived hydrogels. We highlight some opportunities for expanding research incorporating lung ECM-derived hydrogels and potential improvements for the current approaches. Expanding the capabilities of investigations using lung ECM-derived hydrogels is positioned at a cross roads of disciplines, the path to new and innovative strategies for unravelling disease underlying mechanisms will benefit greatly from interdisciplinary approaches. While challenges need to be addressed before the maximum potential can be unlocked, with the rapid pace at which this field is evolving, we are close to a future where faster, more efficient and safer drug development targeting the disrupted 3D microenvironment is possible using lung ECM-derived hydrogels.

## 1 Introduction

The human body is a complex, dynamic environment, consisting of many different cell types that reside in or traverse through defined microenvironments, which is tightly regulated to maintain a healthy state. When elements within this system become disrupted, this can lead to the development of disease. In the lung, disruption of the complex interplay between cells and the extracellular matrix (ECM), the scaffold that provides support and biochemical and biomechanical cues, is emerging as a key element for deciphering the mechanism underlying diseases.

### Why should we think about 3D in *in vitro* model systems?

When we think about the lung *in vivo*, we readily acknowledge that it is a flexible, relatively soft tissue that is three dimensional (3D) in structure. However, in general, when we work with model systems *in vitro*, to try to elucidate processes that underlie homeostasis and disease, we mostly work with two dimensional (2D) systems. In the lung cells are surrounded by a specialised ECM, that is appropriate for their location. Mesenchymal cells are located within a 3D ECM structure, while epithelial and endothelial cells are usually attached to a basement membrane on their basal side and their apical side is subjected to flow of epithelial lining fluid or blood respectively. The stiffness of the lung tissue, in health is usually between 1–5 kilo-pascals (kPa), and in fibrotic disease this can increase up to 100 kPa although the pattern of stiffness can be very heterogeneous (Booth et al., 2012; de Hilster et al., 2020). 2D systems are frequently based on a tissue culture plastic or glass surface, with a stiffness in the gigapascal range, and all cells are grown with polarity. While a lot has been gained from working in 2D systems there is now an opportunity to move forward with our models to establish cells in an environment that reflects the physiological conditions in the lung.

The literature builds a strong body of evidence that the microenvironment in which a cell resides dictates its responses. From simple single ECM component studies (Hirst et al., 2000; Freyer et al., 2001; Bonacci et al., 2003; Parameswaran et al., 2004; Nguyen et al., 2005; Peng et al., 2005; Bonacci et al., 2006; Dekkers et al., 2007; Reddel et al., 2013; Morris et al., 2014), through to more complex cell deposited ECM studies (Johnson et al., 2004; Chan et al., 2006; Harkness et al., 2017), the influence of the ECM components on lung cell proliferation, migration, factor output and response to treatment is evident. However, this information has been collated from cells exposed to ECM components in 2D. It is recognised that cells in a 3D environment have differential responses compared to those in 2D (Duval et al., 2017; Jensen and Teng, 2020). Therefore, developing systems where the influence of the ECM and the microenvironment in 3D can be explored will represent a next step forward for understanding disease underlying mechanisms in the lung.

### Hydrogels from synthetic vs. natural materials

When considering the possibilities for generating 3D microenvironments in which lung cells can prosper there are many different options available. Within the tissue engineering field much work has concentrated on the development of polymers from which soft or stiff hydrogels can be cast or 3D printed (Tibbitt and Anseth, 2009; Melchels et al., 2012; Hospodiuk et al., 2017; Gungor-Ozkerim et al., 2018). These synthetic polymers [including polyacrylamide (Marinkovic et al., 2013) and dextran (Matera et al., 2020)] offer many opportunities for tuning biomechanical and structural properties of the microenvironment but are generally inhospitable environments for cells, requiring the addition of cell binding epitopes, such as RGD motifs, to enable cellular attachment (Lutolf and Hubbell, 2005; Reddy et al., 2021; Caracena et al., 2022). Alternatively, natural ECM components have also been used to generate single component hydrogels that readily support cell attachment, but are more limited in the possibilities for tuning their biomechanical properties. Examples of such hydrogels include collagen type I, fibrin, gelatin (methacrylate) and hyaluronan (Bourke et al., 2011; Tjin et al., 2017; Sun et al., 2020; Martinez-Garcia et al., 2021b; Hui et al., 2021; Blokland et al., 2022; Loebel et al., 2022; Martinez-Garcia et al., 2022). Such hydrogels provide the 3D environment for cells, modelling the dimensionality and possibly the biomechanical mimicry of the *in vivo* situation, but they are not reflective of the complexity of the ECM components within the tissue microenvironment. Hydrogels developed from the solubilized basement membrane matrix secreted by Engelbreth-Holm-Swarm (EHS) mouse sarcoma cells (marketed as Matrigel or Geltrex) have been used for more than 35 years to support cell growth for specific assays, particularly focussing on stem cell expansion assays (Kleinman et al., 1982; Kleinman and Martin, 2005; Benton et al., 2009; Hughes et al., 2010). However, not all cells thrive in such an environment and there are limited possibilities to manipulate the composition and biomechanical environment herein.

A recent advance for the lung field has been the development of hydrogels generated from ECM derived from decellularized lungs. Porcine lung ECM-derived hydrogels were initially reported (Pouliot et al., 2016), while human lung ECM-derived hydrogels have recently been established (de Hilster et al., 2020). This perspective article presents the latest advances in lung ECM-derived hydrogels with respect to their development, modification, characterization and utilization. Moreover, it explores opportunities and challenges for the field, highlighting where future research should focus to improve the comparability of data generated with different measurement systems using lung ECM-derived hydrogels. Finally, we discuss the multi-disciplinary nature of the research required to move these model systems forward.

## 2 The possibilities with lung ECM-derived hydrogels

### 2.1 Lung ECM-derived hydrogels for mimicking *in vivo* ECM biochemical composition

The ECM, including in the lung, is a complex structure of proteins, glycoproteins, matricellular proteins and many other regulatory proteins and enzymes that keep this dynamic structure in balance during tissue homeostasis (Hynes and Naba, 2012). Mimicking such a complex structure when generating an *in vitro* 3D environment in which to culture cells to study cell:matrix interactions is impossible when starting with individual components. Sourcing the ECM from decellularized lungs has provided an opportunity to develop hydrogels that reflect a major proportion of the elements within this complex mixture. The process of decellularization requires treating the tissue with a range of detergents and/or salt solutions (Booth et al., 2012; Wagner et al., 2014; Dabaghi et al., 2021) that do remove some of the elements that are part of the matrisome, particularly growth factors bound to the ECM and some glycoproteins, but the major structural fibres are retained during this process.

Early proteomic studies (Booth et al., 2012; Uhl et al., 2018) illustrated the retention of many components of the lung matrisome in decellularised scaffolds from control and diseased lung samples. A recent study from the team in the Weiss lab (Hoffman et al., 2023), has elegantly shown that the components of the ECM are specific for different compartments within the lung (airways, alveolus, blood vessels), and that these change during chronic lung disease. These decellularized scaffolds from lung tissues have now been used as a source of ECM for the generation of hydrogels. The processing of the scaffolds to generate the solution that will gel when brought to physiological conditions is not thought to lead to further loss of ECM components, making this an ideal method for developing a 3D *in vitro* model system in which cells can be cultured in the presence of this complex mixture of the lung ECM microenvironment.

**FIGURE 1:**
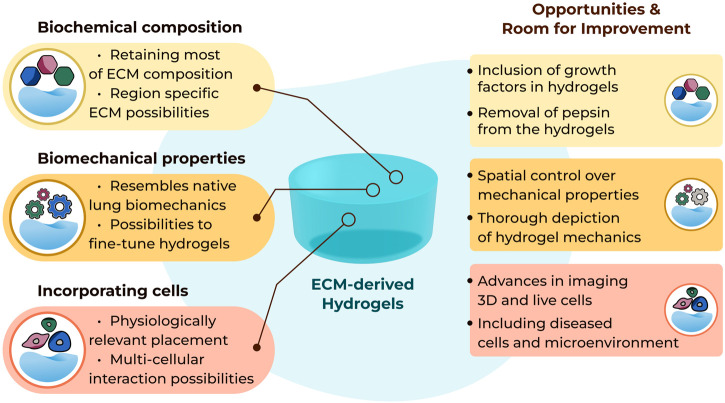
Summary of current possibilities and future opportunities with three-dimensional lung ECM-derived hydrogels.

It remains to be seen if the absence of the elements of the matrisome that are lost during the decellularisation process impose a limitation in the interpretation of data generated when cells are seeded in such hydrogels (Figure 1). The absence of growth factors anchored in the ECM scaffolds, and therefore the ECM-derived hydrogels, after the decellularization process may be considered a limitation, although it is evident that the growth factor retentive properties of the ECM are retained as growth factors supplied in growth media or as part of the secretome from other cells are rapidly absorbed and then subsequently released from the ECM hydrogels (van Dongen et al., 2019). In addition, ECM-derived hydrogels are a source of extracellular vesicles (Ulldemolins et al., 2022), adding another aspect to the regulatory processes induced by these cell support structures.

### 2.2 Lung ECM-derived hydrogels for mimicking tissue biomechanical environments

The structural environment provided for cells by lung ECM-derived hydrogels is another advantage when aiming to develop *in vivo* mimicking model systems. Although the adoption of the method for generating lung ECM-derived hydrogels was only recently reported (Pouliot et al., 2016; de Hilster et al., 2020), the field is advancing rapidly with innovative approaches exploring how different properties can be measured and modified. Among these properties, mechanical properties and topography are two important characteristics of the hydrogels.

When considering mechanical properties of the hydrogels stiffness, Young’s modulus, viscosity or viscoelastic stress relaxation are the usual parameters measured (Vedadghavami et al., 2017). To date, a number of different strategies for measuring mechanical properties of lung ECM-derived hydrogels have been described, although it is important to highlight the challenge when it comes to comparing different studies performed using different measurement approaches for the mechanical properties (Polio et al., 2018). **Rheometry** is one of the most commonly applied methods for measuring mechanical properties of hydrogels (Stojkov et al., 2021). So far, characterization using rheometry has been applied to measure storage (G’) and loss (G”) moduli of porcine (Pouliot et al., 2016) and human lung ECM-derived hydrogels (Dabaghi et al., 2021). In addition, viscosity and Young’s modulus of porcine-sourced lung hydrogels were reported using parallel plate rheometry (Falcones et al., 2021). Other studies have utilized this method on alginate-porcine ECM (De Santis et al., 2021), poly(ethylene glycol) (PEG)-murine ECM (Saleh et al., 2022), and PEG-porcine ECM (Petrou et al., 2020) hybrid hydrogels. **Low-Load compression testing (LLCT)** is another compression-based method (Sharma et al., 2011) that has been used with lung ECM-derived hydrogels. Stiffness and viscoelastic stress relaxation capacity of human non-disease control, chronic obstructive pulmonary disease (COPD) and idiopathic pulmonary fibrosis (IPF) lung ECM-derived hydrogels have been reported; moreover, the mechanical properties of the hydrogels derived from these diseased lungs resembled such properties of the native tissues from which the ECMs were sourced (de Hilster et al., 2020). Similarly, LLCT-measured stiffness and stress relaxation parameters of both native and chemically crosslinked porcine lung ECM-derived hydrogels were also reported (Martinez-Garcia et al., 2021a; Nizamoglu et al., 2022a). Lastly, **atomic force microscopy (AFM)**, which is a more micro-level mechanical measurement based on indentation, was recently used to characterize Young’s modulus values of porcine lung ECM hydrogels (Falcones et al., 2021).

Measuring the mechanical properties is not only useful for diseased environment characterization, but also for verification of the success of methodologies designed to alter such properties. While it is clear that the use of different concentrations of the starting ECM material (powder) (Pouliot et al., 2016) and adjusting the pepsin digestion duration (the essential step in generation of a pre-gel ECM-derived substrate) (Pouliot et al., 2020) influences the mechanical properties, one of the initial attempts to specifically modulate the mechanical properties of lung ECM-derived hydrogels was treating the porcine lung ECM with genipin to increase the stiffness (Link et al., 2017). This approach has been extended with thiol-functionalization (Petrou et al., 2020; Saleh et al., 2022), alginate-reinforcing (De Santis et al., 2021) or fibre crosslinking (Nizamoglu et al., 2022a) to allow greater control over mechanical parameters in the lung ECM-derived hydrogels.

The concepts of altering the mechanical properties, measuring and reporting these changes triggered in the lung ECM-derived hydrogels have been evolving as more novel tools are developed (Figure 1). However, mechanical characterization of lung ECM-derived hydrogels is far from completed. As of today, tensile testing or fatigue testing on such hydrogels have yet to be performed, although using polyacrylamide-ECM hybrid hydrogels these properties were characterized in an early study (Sava et al., 2017). A thorough mechanical and cross-platform characterization of lung ECM-derived hydrogels has not been reported yet. Providing (the comparison of) such characterizations would help the field regarding the interpretation and comparison of different studies using different methods to measure similar parameters. As the field is new, establishing different methods and discussing their advantages and limitations will be important for being able to understand the emerging knowledge about ECM mechanical properties and the functional impacts of these microenvironment parameters.

Another important property which goes hand-in-hand with mechanics is topography, reflecting the fibrous landscape within the ECM-derived hydrogels. In chronic lung diseases like COPD or lung fibrosis, the ECM topography is altered next to the mechanical properties of ECM (Abraham and Hogg, 2010; Tjin et al., 2014; Tjin et al., 2017; Burgess and Harmsen, 2022; Nizamoglu and Burgess, 2022). Using lung ECM sourced from diseased human lungs, for the generation of the hydrogels, would inherently convey (most of) the biochemical composition and resemble the mechanical properties; however, the native architecture of the lung ECM assembly is lost during the process of preparing ECM-derived hydrogels. Recently, preparing porcine lung ECM hydrogels with micropatterned surfaces was described as a method to prepare arrays for drug screening (Zhu et al., 2022). This study demonstrates the preparation of spherical patterns on the hydrogel surface with different diameters, although the aspects of altering the hydrogel surface to guide cell fate, behaviour or differentiation remain unexplored. Alternatively, electrospinning could provide another opportunity to alter the structural organization of the fibres (Hasirci and Hasirci, 2018). While electrospun poly(L-lactic acid) (PLLA)/porcine lung ECM hybrid scaffolds have been previously established (Young et al., 2017), there is no report of electrospinning of pure decellularized lung ECM. Developing novel tools to modify the topography of the ECM fibres within the hydrogels and the regulation of the structural arrangements within these hydrogels requires more attention. Surface modifications on non-ECM-derived hydrogels is not a novel concept (Richbourg et al., 2019; Cai et al., 2020), yet little is known about applying such modifications to the locations within the hydrogels in different planes in order to mimic the architecture of the native lung tissue.

The details of measuring, reporting and altering the properties of lung ECM-derived hydrogels gain more importance as the field progresses. Unfortunately to date, attempts at modifying properties of lung ECM-derived hydrogels remain rather limited. While the latest studies have focused on altering mechanical properties at a global level, new and innovative methodologies that will allow us to initiate more targeted modifications in such properties are required. Especially considering the heterogeneity of lung tissue and its architecture, having more control over spatial distribution of alterations in mechanical properties would enhance the *in vivo* mimicking capacity of our models.

### 2.3 Lung ECM-derived hydrogels for mimicking cell:matrix interactions

The *in vivo* mimicry of the composition and mechanics of the cellular microenvironment present in the lung ECM-derived hydrogels creates an ideal setting for culturing cells within a 3D spatial location. As soon as cells are seeded in hydrogels they begin to remodel their microenvironment (Tjin et al., 2017; Martinez-Garcia et al., 2021b; Martinez-Garcia et al., 2022). Early reports of cells in lung ECM-derived hydrogels reflect findings in single component ECM hydrogels (Tjin et al., 2017), indicating that cells remodel the ECM in which they are embedded, and the nature of the ECM that they encounter directs these remodelling events (Nizamoglu et al., 2022b; Falcones et al., 2022). This fact makes the use of lung ECM-derived hydrogels sourced from diseased lungs an ideal model to understand cellular responses within such a diseased microenvironment and to provide greater knowledge of the influence of the microenvironment to treatment effects.

Initial studies using porcine lung ECM-derived hydrogels reported successful growth of human and rat mesenchymal stromal (stem) cells (MSCs) in 2016 (Pouliot et al., 2016). Link et al. (2017) then described successful culture of mouse MSCs, human alveolar epithelial cells (the cell line A549), human primary microvascular endothelial cells (HpuVECs), and human umbilical vein endothelial cells (HUVECs) in porcine lung ECM-derived hydrogels. The field is now rapidly expanding with additional cells types including murine fibroblasts (Petrou et al., 2020), rat lung MSCs (Falcones et al., 2021) and rat primary alveolar epithelial cells (Marhuenda et al., 2022b) being grown in porcine lung ECM-derived hydrogels. The use of human lung ECM-derived hydrogels is now also possible, with human fibroblasts and airway smooth muscle cells being grown both within and on top of these hydrogels (De Santis et al., 2021; Nizamoglu et al., 2022a; Nizamoglu et al., 2022b).

The field is now moving forward with the cellular systems that are being explored, taking advantage of the values of lung ECM-derived hydrogels. Multi-cellular culture systems are being developed to enable cellular cross-talk in a 3D microenvironment to be examined (Park et al., 2018), and lung ECM-derived hydrogels are being incorporated into other experimental systems (for example, lung on chip or stretching/mechanical force setups) to bring the cell microenvironment in those systems also (Park et al., 2021; Marhuenda et al., 2022a). The possibilities for 3D printing lung ECM-derived hydrogels are also being examined, suggesting greater scope for spatial arrangement of cells within their 3D microenvironment will be possible in the future (De Santis et al., 2021; Falcones et al., 2021).

While the 3D model systems made possible with the use of lung ECM-derived hydrogels are rapidly advancing, the readouts that can be used to investigate end points within these systems are presenting some limitations (Figure 1). Traditional imaging setups are excellent for capturing images in 2D but moving into the third dimension proves challenging to visualise. Lung ECM-derived hydrogels are not translucent, like many of the single ECM or synthetic hydrogels, and this opacity challenges the optical depth of field. The autofluorescence of the lung ECM generates a very noisy image when using many traditional fluorescent reporters. Finally, tracking cell behaviours over time in 3D is extremely difficult to automate when the cells continuously move out of the plane of focus. Advances in imaging and capturing information from cells when they are interacting within their microenvironment is urgently needed to facilitate the full capacity of lung ECM-derived hydrogels.

## 3 Discussion and future remarks

Studies in lung ECM-derived hydrogels will help to inform us of the optimal microenvironment for different cell types, as the cells continuously remodel their environment in, what appears to be, a programmed response. Whether there is temporal regulation of the remodelling, in particular in response to injury, is an outstanding question for the field. How these processes are altered in chronic lung diseases, and whether the progression of such processes can be reversed is knowledge that can be informed through the use of lung ECM-derived hydrogels. The approaches described above, including modulating mechanical properties of ECM-derived hydrogels without changing the ECM composition and the application of mechanical forces to cells within a 3D microenvironment, are attractive as they will facilitate research enabling the field to begin separating influences of the mechanical changes from those of the biochemical changes in the ECM in lung diseases. Such elucidation may open the door for development of mechanosensitive therapeutic targets for lung diseases.

**FIGURE 2:**
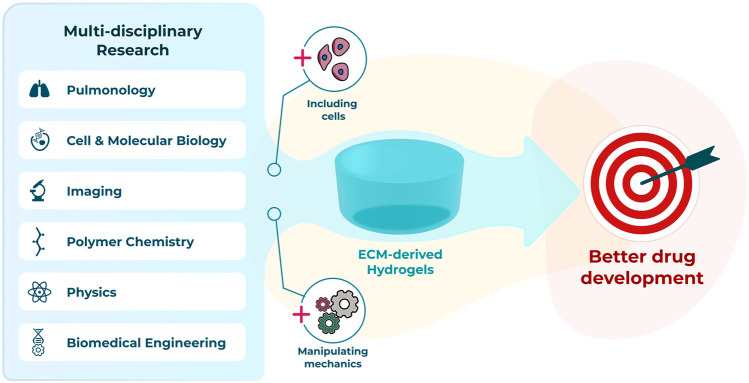
Interdisciplinary advances to progress towards a better preclinical model using hydrogels.

To fully leverage the advantages offered by lung ECM-derived hydrogels multi-disciplinary teams who bring together expertise from the diverse fields needed to advance such systems will be necessary (Figure 2). Innovative researchers from pulmonology, cell and molecular biology, polymer chemistry, biomedical engineering, imaging and physics backgrounds are all needed to maximise opportunities and ensure the current challenges quickly become advantages for this exciting, emerging area of lung disease research.

## Data Availability

The original contributions presented in the study are included in the article/Supplementary Material, further inquiries can be directed to the corresponding author.
